# A Bibliometric and Systematic Review of Quantitative Microbial Risk Assessment in Food Safety (1995–2024)

**DOI:** 10.3390/foods15071197

**Published:** 2026-04-02

**Authors:** Amil Orahovac, Nađa Raičević, Aleksandra Martinović, Werner Ruppitsch, Robert L. Mach

**Affiliations:** 1 Faculty of Food Technology, Food Safety and Ecology, University of Donja Gorica, 81000 Podgorica, Montenegro; nadja.raicevic@udg.edu.me (N.R.); aleksandra.martinovic@udg.edu.me (A.M.); werner.ruppitsch@i-med.ac.at (W.R.); 2 Institute of Hygiene and Medical Microbiology, Medical University Innsbruck, 6020 Innsbruck, Austria; 3 Institute of Chemical, Environmental and Bioscience Engineering, Research Area of Biochemical Technology, TU Wien, 1060 Vienna, Austria

**Keywords:** quantitative microbial risk assessment, QMRA, food safety, bibliometric analysis, probabilistic modelling, exposure assessment, predictive microbiology, foodborne pathogens

## Abstract

Quantitative microbial risk assessment (QMRA) has become a central framework for evaluating foodborne microbial hazards by integrating microbiological data, exposure assessment, dose–response modelling, and probabilistic simulation. Over the past three decades, its rapid expansion has created challenges in obtaining a coherent overview of the field’s structure, dominant themes, and research trajectories. This study presents a bibliometric and systematic review of QMRA research in food safety. Bibliographic data were retrieved from the Scopus database (search conducted in January 2026), including peer-reviewed articles published in English between 1995 and 2024, and analysed using performance analysis and science mapping techniques to assess publication trends, influential contributors, collaboration patterns, and thematic evolution. Risk of bias assessment was not applicable due to the bibliometric nature of the study. The results indicate steady long-term growth of QMRA research, based on a final dataset of 186 articles across multiple journals and countries, with a concentrated influence structure dominated by a limited number of specialised journals, institutions, and research groups. International collaboration is particularly strong within European networks. Thematic analysis identifies probabilistic exposure assessment, Monte Carlo simulation, predictive microbiology, and dose–response modelling as the methodological core, with a primary focus on major foodborne pathogens such as *Campylobacter*, *Salmonella*, *Listeria monocytogenes*, and *Escherichia coli*. Persistent emphasis on uncertainty, cross-contamination, and dose–response relationships highlights key methodological challenges. Limitations include reliance on a single database and potential exclusion of studies using alternative terminology. These findings provide a structured overview of the QMRA landscape and identify priorities for methodological refinement and future application in food safety risk assessment. This study received no external funding and was not prospectively registered.

## 1. Introduction

The foodborne diseases caused by microbial hazards remain a persistent global public health challenge, despite substantial advances in food safety management systems, microbiological criteria, and process control strategies. Pathogens such as *Listeria monocytogenes*, *Salmonella* spp., *Campylobacter* spp., Shiga toxin–producing *Escherichia coli* (STEC), and norovirus continue to be associated with sporadic cases, outbreaks, and severe clinical outcomes, particularly among vulnerable populations. The complexity of modern food supply chains, variability in production and consumption practices, and inherent uncertainty in microbial behaviour limit the effectiveness of purely descriptive or qualitative risk management approaches. As a result, there has been a sustained need for analytical frameworks capable of translating microbiological contamination data into public-health-relevant risk estimates [1,2].

Quantitative microbial risk assessment (QMRA) emerged in the mid-1990s as a response to this need, adapting probabilistic risk assessment principles to microbial food safety contexts. Unlike the more straightforward assessment of chemical contaminants, microbial risk is inherently more complex to quantify. The difficulty arises from several factors: microbial systems are highly dynamic, making their behaviour hard to predict, and the tools used for detection can vary widely, sometimes identifying even dead cells [3]. This variability further complicates accurate risk assessment. Early conceptual work established QMRA as a structured framework linking hazard identification, exposure assessment, dose–response modelling, and risk characterisation, thereby enabling estimation of the probability and magnitude of adverse health outcomes associated with foodborne pathogens [1,2]. Unlike qualitative or semi-quantitative approaches, QMRA explicitly accounts for biological variability and parameter uncertainty and allows evaluation of alternative scenarios, interventions, and management options through probabilistic simulation [4]. Foundational contributions highlighted the central role of Monte Carlo simulation and predictive microbiology in bridging contamination data and health outcomes [5].

Methodologically, QMRA integrates heterogeneous data sources spanning primary production, processing, distribution, retail, and consumer handling. Exposure assessment is typically constructed as a modular or farm-to-fork model in which microbial prevalence, concentration, growth, inactivation, and cross-contamination are represented as stochastic processes [5]. Predictive microbiology models are embedded to describe pathogen behaviour under varying environmental conditions, while dose–response models translate ingested doses into probabilities of infection or illness [6]. Probabilistic techniques, most commonly Monte Carlo simulation, are used to propagate variability and uncertainty throughout the exposure chain and to generate quantitative risk distributions [7]. Over time, methodological refinements have addressed issues such as left-censored data, sparse datasets, sensitivity analysis, and alternative modelling frameworks, including Bayesian approaches, to support inference under data-limited conditions [7,8].

Since its conceptual consolidation, QMRA has transitioned from a primarily academic methodology to a decision-support tool increasingly used by regulatory agencies and advisory bodies. Early end-to-end applications demonstrated the feasibility of dynamic exposure modelling and stochastic simulation for pathogens such as *E. coli* O157:H7 in beef products [5]. Subsequent studies extended the approach to a wide range of food–pathogen combinations, notably *Listeria monocytogenes* in ready-to-eat foods [9,10,11], *Salmonella* in poultry, eggs, and low-water-activity foods [8], *Campylobacter* in poultry products [12], and viral hazards in seafood [13]. These applications illustrated how QMRA outputs can be used to evaluate intervention strategies, inform microbiological criteria, and compare risk management options. In parallel, international organisations and national authorities, including European food safety institutions, have incorporated QMRA into scientific opinions and regulatory risk assessments, further reinforcing its policy relevance [4,14].

Despite these advances, the QMRA literature consistently identifies unresolved challenges that constrain wider applicability and comparability across studies. Data availability and quality remain limiting factors, particularly for consumer behaviour, post-retail handling, and traditional or small-scale food systems [15,16]. Dose–response relationships for several pathogens remain uncertain, and methodological heterogeneity complicates cross-study comparison and harmonisation [6,17]. Moreover, the integration of emerging data sources, such as genomic information or advanced modelling techniques, into QMRA frameworks remains uneven [17,18]. These challenges have stimulated ongoing methodological development and have contributed to a rapidly expanding and increasingly diverse body of QMRA literature.

Given this growth, a systematic synthesis of the QMRA research landscape is needed to understand how the field has evolved, which methodological and thematic directions dominate current research, and where critical gaps persist. While narrative and systematic reviews have addressed specific pathogens or commodities, a comprehensive bibliometric perspective can provide complementary insights by quantitatively mapping publication trends, intellectual influence, collaboration structures, and thematic evolution across the field. Bibliometric analysis offers a transparent and reproducible means of exploring large volumes of scientific literature, enabling the identification of dominant research clusters, leading contributors, and emerging topics that may not be readily apparent through traditional review approaches. Although the final dataset comprised a moderate number of records, bibliometric analysis was considered the most appropriate design because the objective of this study was not to synthesise effect estimates, compare intervention outcomes, or aggregate quantitative risk metrics across QMRA applications. Instead, the aim was to examine the structural properties of the field itself, including publication dynamics, intellectual influence, collaboration patterns, and thematic evolution. These dimensions concern how scientific knowledge is produced, disseminated, and organised rather than what individual studies report. Bibliometric performance analysis and science mapping are therefore better suited to address such structural and relational questions than a conventional systematic review focused on outcome synthesis.

Accordingly, the present study applies bibliometric methods to systematically analyse the scientific literature on quantitative microbial risk assessment in food safety. By examining publication dynamics, influential authors and journals, international collaboration patterns, and keyword-based thematic structures, this work aims to characterise the conceptual, social, and intellectual foundations of QMRA research. In doing so, it provides an evidence-based overview of how QMRA has developed over time, where research efforts are currently concentrated, and which methodological and application areas warrant further investigation.

Specifically, this study addresses five research questions (RQs):

1. How has QMRA research output evolved over time between 1995 and 2024?
2. Which journals and publications have exerted the greatest scientific influence within QMRA research?
3. Which authors and institutions have played a central role in shaping the field?
4. How is QMRA research distributed geographically and structured through international collaboration?
5. Which pathogens, methodological components, and thematic areas dominate the literature and indicate emerging research directions?

## 2. Materials and Methods

**Figure 1:**
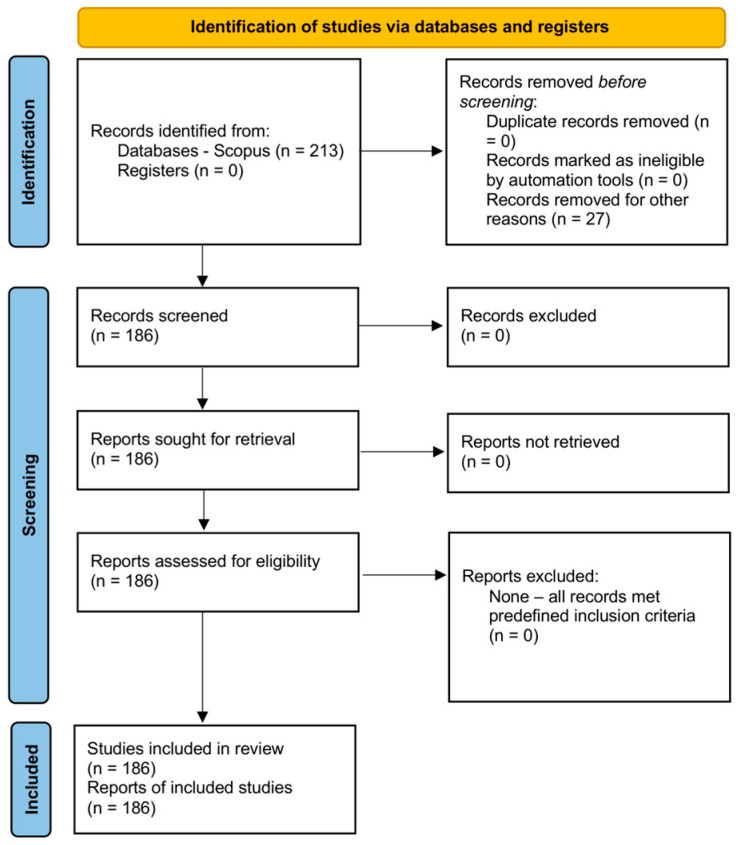
PRISMA 2020 flow diagram of the study selection process.

A bibliometric analysis was conducted to systematically map the scientific literature on quantitative microbial risk assessment (QMRA) applied to foodborne hazards. This study did not aim to synthesise effect estimates or health outcomes, but to map the structure, influence, and thematic evolution of QMRA research. Unlike narrative or traditional systematic reviews, which rely primarily on qualitative interpretation, bibliometric analysis applies quantitative, reproducible methods that enable transparent exploration of large bodies of scientific literature [19]. This approach allows the identification of publication trends, influential contributors, dominant research themes, and collaboration patterns within a research field in a consistent and replicable manner. It examined the temporal development and growth dynamics of quantitative microbial risk assessment (QMRA) research over the period 1995–2024. The analysis period began in 1995, which corresponds both to the earliest indexed QMRA-related publications in Scopus and to the initial emergence of QMRA as a distinct methodological approach in food safety research. The study design and reporting were conducted in accordance with the PRISMA 2020 guidelines for systematic reviews [20]. Although the analytical component of the study is bibliometric in nature, the literature identification process followed a structured and transparent workflow consistent with PRISMA recommendations. Bibliographic records were retrieved from the Scopus database using a predefined search strategy. Following initial retrieval (*n* = 213), eligibility filters related to publication year, document type (articles only), and language (English) were applied, resulting in a final dataset of 186 records. No records were excluded during the screening stage, as all remaining studies were confirmed to meet the predefined inclusion criteria. Eligibility screening was conducted independently by two reviewers (first author and senior author) based on titles, abstracts, and, where needed, full texts. Any discrepancies were resolved through discussion and consensus. Given the specificity of the search strategy and applied filters, all records remaining after initial filtering met the inclusion criteria and were retained. No automation tools were used in the selection process. The complete selection process is illustrated in the PRISMA 2020 flow diagram (Figure 1), and the completed PRISMA checklist is provided in the Supplementary Materials.

The review was not prospectively registered. Prospective registration is more commonly required for systematic reviews that synthesise intervention effects or clinical outcomes, whereas bibliometric studies focus on structural and relational characteristics of scientific literature. Nevertheless, we acknowledge that prospective registration could have further enhanced methodological transparency by documenting the analytical framework in advance.

All analyses were performed using the Bibliometrix package (version 5.2.1) [21] in the R statistical environment [22], which provides established tools for performance analysis and science mapping of bibliographic data.

### 2.1. Data Source and Search Strategy

Bibliographic data were retrieved from the Scopus (Elsevier, Amsterdam, The Netherlands) database, selected for its broad coverage of peer-reviewed journals and standardized bibliographic metadata. Scopus was used as the sole database because it provides extensive coverage of international scientific literature, consistent indexing of bibliographic fields required for bibliometric mapping, and compatibility with bibliometrix workflows. In addition, the use of a single database reduces duplication, indexing inconsistencies, and record harmonization issues that may arise when combining sources with different metadata structures. Nevertheless, reliance on a single database may have led to the omission of relevant studies indexed elsewhere, which is acknowledged as a limitation of the study. The search was conducted on 24 January 2026. Although the search was conducted in January 2026, the analysis was restricted to publications indexed up to the end of 2024. The year 2025 was intentionally excluded to avoid distortion caused by incomplete database coverage, ongoing indexing, and partial citation accumulation for the most recent year. Literature retrieval was performed using a structured search strategy applied to titles, abstracts, and author keywords (TITLE-ABS-KEY). Boolean operators, phrase searching, and wildcard operators were used to ensure comprehensive coverage of relevant terminology.

The search query used in this study is presented in Listing 1:

**Listing 1.** Scopus search query used for identifying QMRA-related publications.TITLE-ABS-KEY ( “quantitative microbial risk assessment” OR “QMRA” OR ( “microbial risk assessment” AND ( quantitative OR probabilistic OR stochastic ) ) OR ( “quantitative risk assessment” AND microbial ) ) AND TITLE-ABS-KEY ( Listeria OR Salmonella OR Campylobacter OR “*Escherichia coli*” OR “E. coli” OR STEC OR Norovirus ) AND TITLE-ABS-KEY ( “dose-response” OR “exposure assessment” OR “predictive microbiology” OR “Monte Carlo” OR “probabilistic model*” OR “stochastic model*” ) AND TITLE-ABS-KEY ( food OR “ready-to-eat” OR meat OR dairy OR seafood OR poultry OR produce OR vegetable* OR fruit* ) AND PUBYEAR > 1994 AND PUBYEAR < 2025 AND ( LIMIT-TO ( DOCTYPE, “ar” ) ) AND ( LIMIT-TO ( LANGUAGE, “English” ) )

The initial search returned 213 records. After applying predefined eligibility filters related to publication year (1995–2024), document type (articles only), and language (English), 27 records were excluded prior to screening, resulting in a final dataset of 186 studies included in the bibliometric analysis. This search strategy was designed to explicitly capture studies applying quantitative and probabilistic microbial risk assessment frameworks, with emphasis on dose–response modelling, exposure assessment, and stochastic or Monte Carlo-based approaches in food safety contexts. The temporal window was selected to reflect the emergence and maturation of QMRA as a distinct methodological field. Bibliographic data were exported from Scopus in a structured format and included information on authors, affiliations, source titles, publication year, citations, and author keywords. Data preparation involved standardization of author names, institution names, and keywords to ensure consistency in subsequent analyses. No additional data were requested from study authors.

### 2.2. Data Screening and Inclusion Criteria

Only peer-reviewed research articles published in English between 1995 and 2024 were included. Studies were required to explicitly address quantitative microbial risk assessment (QMRA) or closely related probabilistic microbial risk modelling approaches in food systems. Publications not meeting these criteria, including non-English documents, non-article document types, and studies outside the defined time frame, were excluded through database filtering prior to screening. Title and abstract screening was conducted independently by two reviewers to verify eligibility and confirm thematic relevance. No records were excluded during this stage. This outcome reflects the deliberately narrow and concept-driven search strategy, which combined QMRA-specific terminology with pathogen-, modelling-, and food-related keywords. As a result, records remaining after database filtering were already highly specific to the study scope, and the screening stage functioned primarily as an eligibility confirmation step rather than a major exclusion phase. All included studies were considered eligible for bibliometric analysis and were analysed collectively without subgroup-based synthesis. Inclusion criteria were defined based on study design (peer-reviewed articles), topic relevance (explicit QMRA or probabilistic microbial risk assessment), language (English), and time frame (1995–2024). No restrictions were applied regarding geographic region or specific food matrices.

### 2.3. Bibliometric Analysis

The bibliographic dataset was analysed using two complementary bibliometric approaches: performance analysis and science mapping.

Performance analysis was used to describe the productivity and impact of the field by examining publication output and citation patterns across journals, authors, institutions, and countries. Descriptive indicators such as publication counts and total citations were used to identify leading sources and contributors within the QMRA literature. Citation-based indicators were derived from the Scopus database metadata and therefore include self-citations unless otherwise specified by the database. Because the primary objective of this study was to characterise the structural and relational properties of the QMRA research landscape rather than to evaluate individual scholarly performance, citation counts were retained in their original form to ensure consistency across performance metrics. However, the inclusion of self-citations should be considered when interpreting influence-related indicators.

Science mapping techniques were applied to explore the conceptual and social structure of the field. Conceptual structure was examined through keyword frequency and co-occurrence analysis based on author keywords, allowing identification of dominant research themes and methodological focuses. Social structure was explored using country-level collaboration networks derived from co-authorship information, highlighting patterns of international scientific cooperation. Prior to analysis, bibliographic data underwent a structured cleaning and standardisation process to improve consistency across performance and network analyses. Author name disambiguation was performed using a combination of bibliometrix harmonisation functions and manual verification to resolve variations in initials, name order, and spelling. Institutional names were standardised by merging variant naming formats referring to the same organisation. Author keywords were reviewed to consolidate synonymous terms and harmonise plural–singular variations where appropriate. This combined manual and tool-assisted approach ensured greater consistency of bibliographic entities and improved the reliability of collaboration and thematic mapping.

All networks and visualizations were generated within the R environment (version 4.5.1/2025-06-13) using Bibliometrix-compatible workflows to ensure transparency and reproducibility of the analytical process. As this study is based on bibliometric analysis of published literature rather than synthesis of experimental or clinical outcomes, outcome measures, effect size estimation, risk of bias assessment, reporting bias assessment, heterogeneity analysis, sensitivity analysis, and certainty of evidence evaluation were not applicable.

## 3. Results and Discussion

The included studies (*n* = 186) represent peer-reviewed QMRA research published between 1995 and 2024 across 46 scientific journals and multiple geographic regions. The dataset encompasses studies focusing on major foodborne pathogens—particularly *Campylobacter*, *Salmonella*, *Listeria monocytogenes*, and *Escherichia coli*—and includes methodological components such as exposure assessment, dose–response modelling, predictive microbiology, and probabilistic simulation.

A detailed list of all included studies, including bibliographic characteristics (authors, year, journal, and keywords), is provided in Supplementary Table S1.

Of the 27 records excluded prior to screening, all were removed based on predefined filters (non-article document types or non-English publications). No records were excluded after screening, as all remaining studies were confirmed to meet the predefined inclusion criteria. No full-text articles that met the inclusion criteria were excluded after screening; therefore, an excluded-studies list is not applicable.

### 3.1. Research Question 1: Temporal Evolution and Growth Dynamics of QMRA Research

This research question examined the temporal development and growth dynamics of quantitative microbial risk assessment (QMRA) research over the period 1995–2024. The final dataset comprised 186 peer-reviewed articles published across 46 scientific journals, reflecting a specialized yet internationally distributed research domain. The average document age was 10.9 years, indicating a mature and continuously reused knowledge base. Citation performance was robust, with an average of 35.1 citations per document and 2.59 citations per year per document, highlighting the sustained scientific relevance of foundational methodological contributions and applied modelling studies.

Overall publication activity exhibited a steady long-term growth trajectory, with an estimated annual growth rate of 8.95%. Early QMRA research emerged sporadically in the late 1990s, characterized by seminal methodological contributions that established dose–response modelling, stochastic exposure assessment, and predictive microbiology as the analytical backbone of quantitative microbial risk analysis. During the early 2000s, annual publication output remained modest, typically limited to fewer than five articles per year, reflecting the substantial data requirements, computational complexity, and interdisciplinary expertise needed to operationalize quantitative microbial modelling.

A gradual consolidation phase became evident after 2010, with annual publication counts stabilizing between five and ten articles per year. This period corresponds to wider adoption of Monte Carlo simulation frameworks, improved microbial growth and inactivation models, and increasing integration of QMRA concepts into applied food safety research and regulatory assessments. The most pronounced expansion occurred after 2015, when annual output consistently exceeded ten publications per year, reaching a peak of 16 articles in 2020. The publication peak observed in 2020 coincides with the onset of the COVID-19 pandemic, which intensified global attention toward infectious disease modelling, risk analysis, and food-system resilience. Although QMRA focuses specifically on foodborne microbial hazards, the broader scientific emphasis on quantitative risk modelling during this period may have indirectly stimulated research activity in the field. However, the subsequent stabilization of publication output suggests that the pandemic did not fundamentally alter the long-term development trajectory of QMRA, but rather may have temporarily amplified interest in quantitative risk-based approaches. This interpretation should be considered cautiously, as bibliometric analysis does not allow direct causal attribution. From 2021 to 2024, publication levels remained relatively stable at approximately 11–12 articles per year, suggesting a transition from rapid methodological expansion toward a mature phase characterized by incremental refinement, diversification of application domains, and consolidation of best practices.

In contrast to the rapid expansion observed in some chemical risk assessment domains, QMRA demonstrates a more controlled and methodologically constrained growth profile. This pattern is consistent with the high technical barriers associated with data acquisition, model validation, uncertainty propagation, and interdisciplinary integration required for robust quantitative microbial risk assessments. The observed temporal trajectory therefore reflects both the maturation of computational and modelling capacities and the progressive institutionalization of QMRA within food safety research, regulatory science, and quantitative decision-support frameworks.

**Figure 2:**
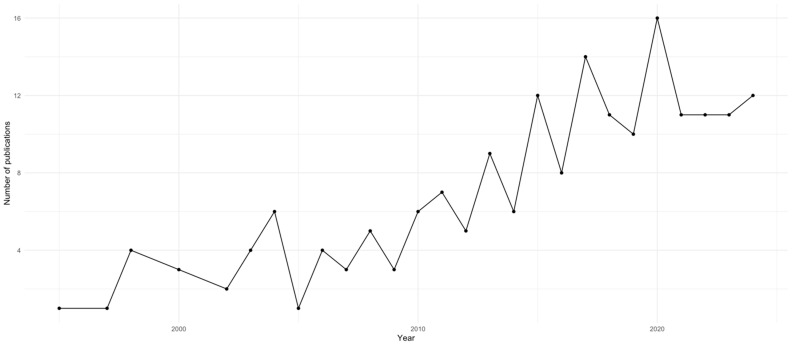
Annual Growth of Publications in Quantitative Microbial Risk Assessment (QMRA) from 1995 to 2024.

Figure 2 illustrates the annual scientific production of QMRA publications over the analysed period, highlighting the gradual emergence of the field, its consolidation phase, and the subsequent stabilization of publication output in recent years.

### 3.2. Research Question 2: Which Journals and Publications Have Exerted the Greatest Scientific Influence Within QMRA Research?

**Figure 3:**
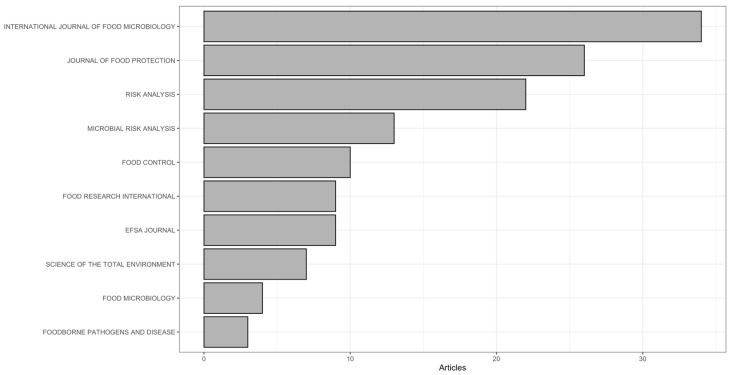
Distribution of QMRA Publications Across Leading Journals (1995–2024).

Journal analysis confirms a strong concentration of QMRA publications within a limited number of specialised outlets (Figure 3).

The International Journal of Food Microbiology was the dominant publication venue (34 articles), followed by the Journal of Food Protection (26) and Risk Analysis (22). A second tier of journals included Microbial Risk Analysis (13), Food Control (10), and both EFSA Journal and Food Research International (9 each), while the remaining outlets contributed fewer than eight articles each. This distribution indicates that QMRA research is anchored in a small set of core journals that collectively shape methodological development and applied risk assessment practice, with additional dispersion into environmental and water-focused journals (e.g., Science of the Total Environment (7) and Water Research (3)), reflecting the gradual expansion of QMRA approaches beyond traditional food-safety settings.

While journals such as Food Control (*n* = 10; total citations = 235), EFSA Journal (*n* = 9; total citations = 38), and Food Research International (*n* = 9; total citations = 163) contribute a moderate number of QMRA papers, they remain key outlets for operational and regulatory-facing work. In contrast, a smaller set of interdisciplinary journals shows disproportionately high citation visibility relative to publication volume—Science of the Total Environment (*n* = 7; total citations = 297; 42.4 citations/article), Water Research (*n* = 3; total citations = 254; 84.7 citations/article), and Environmental Science & Technology (*n* = 2; total citations = 135; 67.5 citations/article). This pattern supports the view that QMRA approaches and findings increasingly diffuse into broader environmental and water risk frameworks, attracting attention beyond traditional food-safety audiences. Knowledge dissemination follows a similarly structured pattern, with a small set of journals serving as the primary channels through which methodological advances and applied case studies circulate. The dominance of the *International Journal of Food Microbiology* highlights the continued centrality of microbiological process understanding, while the strong citation impact of *Risk Analysis* reflects the enduring influence of foundational probabilistic and modelling concepts. At the same time, the presence of journals such as *Food Control*, *EFSA Journal*, and interdisciplinary outlets including *Science of the Total Environment* signals an ongoing translation of QMRA from specialized modelling contexts toward regulatory implementation and cross-sectoral applications.

### 3.3. Research Question 3: Which Authors and Institutions Have Played a Central Role in Shaping the Field?

The influence structure of quantitative microbial risk assessment (QMRA) research reveals a highly interconnected yet thematically structured scientific community, dominated by a relatively small number of prolific authors, specialized institutions, and core journals.

Author productivity analysis identifies Nauta M (14 publications), Evers EG (8), Garré A (7), Havelaar AH (6), Pouillot R (6), Schaffner DW (6), and Zwietering MH (6) as the most prolific contributors. Co-authorship network analysis demonstrates dense collaboration clusters centered around European research groups, particularly those associated with Dutch, French, Danish, and Spanish institutions, indicating long-term methodological collaboration rather than isolated publication activity.

Beyond productivity, the intellectual influence of these authors is reflected in their highly cited contributions and thematic specialization. Nauta and Havelaar have played a central role in establishing Campylobacter QMRA, cross-contamination modelling in domestic food preparation, and the translation of QMRA outputs into risk-based microbiological criteria and food safety objectives [23,24,25,26]. Evers has contributed extensively to exposure assessment frameworks, including simplified QMRA tools (SQMRA), comparative exposure modelling, and large-scale European assessments of *Salmonella* and ESBL-producing *E. coli* across meat chains [27,28,29].

Garré and Zwietering have driven methodological innovation in variability and uncertainty modelling, introducing multilevel Bayesian approaches and strain-level variability into predictive microbiology and QMRA [30,31]. Their work also bridges emerging topics such as dynamic thermal inactivation modelling and the integration of omics concepts into hazard characterization [32]. Pouillot’s contributions are strongly oriented toward computational infrastructure and probabilistic modelling, including the development of R-based tools for separating variability and uncertainty, comparative risk ranking platforms (FDA-iRISK), and sensitivity analysis in *Listeria* growth modelling [33,34,35]. Pérez-Rodríguez has focused on predictive survival modelling and cross-contamination dynamics in fresh produce and food contact surfaces, providing parameterized models that feed directly into probabilistic exposure assessments [36,37,38]. Schaffner’s work emphasizes farm-to-fork QMRA frameworks, particularly for leafy greens, washing processes, and food service environments [39,40,41].

Overall, the thematic distribution of leading authors illustrates a balance between foundational methodological development (dose–response modelling, uncertainty propagation, stochastic simulation) and applied risk assessment for specific pathogen-food combinations, most prominently *Campylobacter*, *Salmonella*, *Listeria monocytogenes*, and pathogenic *E. coli*.

Institutional analysis highlights several dominant research hubs shaping QMRA development. The National Institute for Public Health and the Environment (RIVM) emerges as the leading institution, followed by the Technical University of Denmark (DTU), University of Bologna, Wageningen University & Research, Université Paris-Est/Maisons-Alfort, and major North American centres such as Ottawa-based research institutes. These institutions are strongly embedded in European regulatory science ecosystems and frequently participate in multi-country projects, reflected by high international co-authorship rates.

The institutional network structure suggests that QMRA knowledge production is driven by a limited number of highly specialized centres with sustained methodological capacity, rather than diffuse, isolated research activity. This concentration supports consistency in modelling paradigms, data harmonization practices, and regulatory alignment, particularly within the European food safety context.

**Figure 4:**
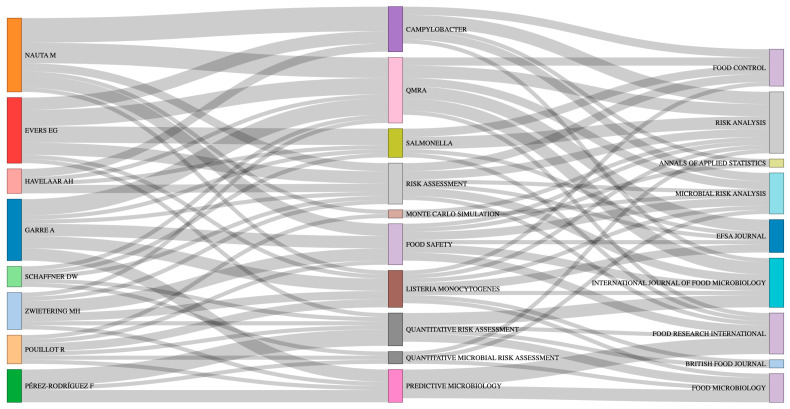
Co-authorship and Thematic Collaboration in QMRA Research (1995–2024).

The Sankey visualization (Figure 4) further illustrates how leading authors preferentially publish within these core journals while simultaneously engaging with distinct thematic clusters, linking methodological innovation with applied risk assessment.

Taken together, the results indicate that QMRA research has evolved into a highly cohesive yet dynamically expanding scientific ecosystem. A relatively small group of prolific authors has shaped the methodological backbone of the field, establishing robust frameworks for exposure assessment, dose–response modelling, uncertainty propagation, and predictive microbiology, while simultaneously translating these methods into applied assessments for priority food–pathogen combinations. Long-term collaborative ties among these researchers, visible in the co-authorship network, suggest sustained methodological continuity rather than fragmented or opportunistic publication patterns.

This intellectual leadership is strongly anchored within a limited number of specialized institutional hubs, particularly in the Netherlands, Denmark, France, Italy, and North America, where regulatory science, academic modelling expertise, and access to harmonized datasets converge. These institutions function not only as centres of publication output but also as generators of transferable modelling practices that shape international risk assessment standards and guidance. The geographic collaboration structure further reinforces this concentration, with dense European networks acting as the backbone of the field, complemented by growing international participation that reflects the globalisation of food systems and the increasing portability of QMRA methodologies across regulatory environments.

The thematic profile of leading authors illustrates how the field balances depth and breadth: core efforts remain focused on major foodborne pathogens such as *Campylobacter*, *Salmonella*, *Listeria monocytogenes*, and pathogenic *E. coli*, yet methodological innovation increasingly targets complex sources of variability, uncertainty, strain heterogeneity, and dynamic process modelling. Emerging connections to omics-based hazard characterization and comparative risk-ranking platforms further suggest a gradual shift toward more integrative and data-intensive risk assessment paradigms.

### 3.4. Research Question 4: Geographic Distribution and International Collaboration Patterns

Research Question 4 examined the geographic distribution of publications and international collaboration patterns in QMRA research, with a particular focus on corresponding author countries, the balance between single-country and multi-country publications, and the structure of international co-authorship networks.

**Figure 5:**
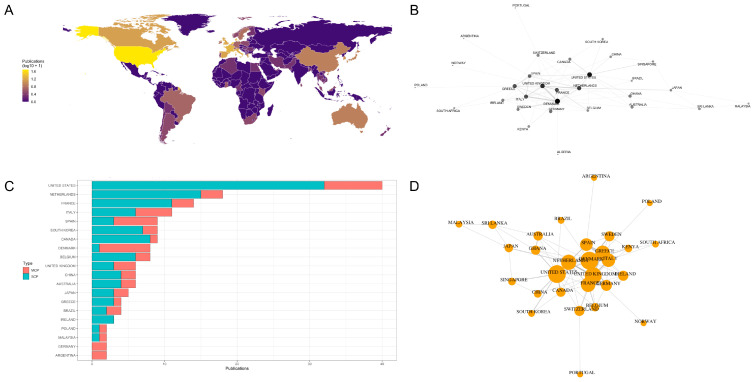
Publication Output, Geographic Distribution, and International Collaboration Networks in Quantitative Microbial Risk Assessment (QMRA) Research (1995–2024). (**A**): Global Distribution of QMRA Publications by Country; (**B**): International Collaboration Network in QMRA Research; (**C**): Leading Countries by Publication Output and Multi-Country Collaboration; (**D**): Country-Level Co-authorship Network in QMRA Research.

The global distribution of publications by country is presented in Figure 5A, which shows a clear concentration of research output in Europe and North America, with additional contributions from East Asia and Oceania. The United States emerged as the leading contributor worldwide, followed by several European countries, including the Netherlands, France, Italy, Spain, Denmark, Belgium, and the United Kingdom. Outside Europe, notable contributions were observed from Canada, South Korea, China, Japan, Australia, and Brazil, while publications from Africa and parts of South America were comparatively sparse. Country-level analysis indicates that the United States, the Netherlands, France, Italy, Spain, Denmark, and Canada dominate publication output and citation impact (Figure 5C). While the United States leads in absolute publication volume and citations, several European countries demonstrate high multi-country publication ratios, reflecting strong transnational collaboration. The Netherlands and Denmark, in particular, function as methodological hubs linking academic research with regulatory implementation.

To further examine authorship patterns, Figure 5D presents the top 20 corresponding author countries, disaggregated into single-country publications (SCP) and multi-country publications (MCP). The United States ranked first in terms of total corresponding author output (40 publications), with a predominance of single-country publications (32 SCP; MCP share = 0.20). Similarly, the Netherlands (18 publications), France (14 publications), and Canada (9 publications) showed relatively low MCP shares, indicating a strong domestic research base. In contrast, several European countries exhibited a substantially higher level of internationalization. Denmark showed the highest MCP share among the top contributors (MCP share = 0.88), followed by Spain (0.67), Italy (0.46), and the United Kingdom (0.50), highlighting the central role of international collaboration in sustaining their research output.

Across the entire dataset, a total of 185 publications were identified at the corresponding author level, of which 120 were single-country publications and 65 were multi-country publications, resulting in an overall MCP share of 0.35. This indicates that more than one-third of the publications in the field involved international collaboration, underlining the transnational nature of QMRA research.

International collaboration patterns are further illustrated in Figure 5D, which depicts the undirected country-level co-authorship network based on collaborative publications. The collaboration network illustrates dense interconnections among European countries, with increasing participation from the Asia-Pacific regions in recent years. This pattern reflects both the globalization of food supply chains and the growing transferability of QMRA methodologies across regulatory contexts.

The network reveals a dense European core, with Denmark, Italy, the United Kingdom, France, and the Netherlands occupying central positions and acting as key hubs connecting multiple partner countries. The strongest bilateral collaboration links were observed between Denmark and Italy, and between Denmark and the United Kingdom (each with a collaboration weight of 6), followed by strong ties between the United Kingdom and France, and between the United Kingdom and the Netherlands (weight = 4). Additional prominent collaboration links included Italy–Spain, Greece–Italy, and Canada–United States, reflecting both regional clustering within Europe and transatlantic cooperation.

Overall, the findings from RQ4 demonstrate that while research output in QMRA is geographically concentrated in a limited number of countries, international collaboration plays a substantial and structurally important role, particularly within the European research area. Countries with smaller national research systems tend to rely more heavily on international co-authorship, whereas larger contributors maintain a stronger balance between domestic and collaborative research.

### 3.5. Research Question 5: What Are the Major Research Themes and Potential Future Research Directions in QMRA and Food Risk Assessment Research?

**Figure 6:**
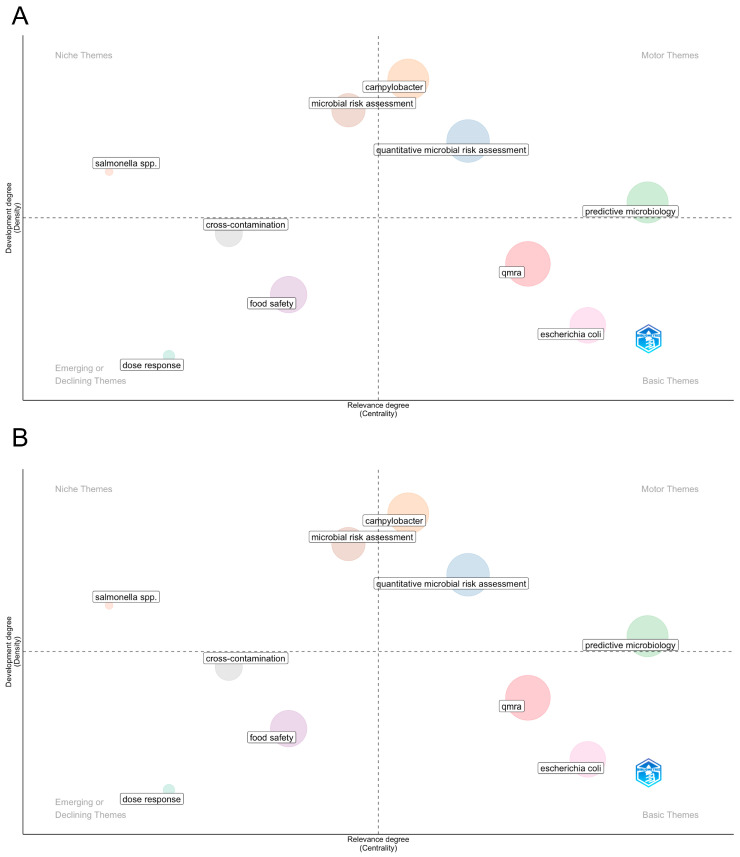
Thematic Mapping of QMRA Research Areas and Emerging Trends (1995–2024): (**A**): Thematic Mapping of Core and Emerging Themes in QMRA Research; (**B**): Development and Centrality of Key Research Themes in QMRA.

The thematic structure of QMRA and food risk assessment research was explored through keyword co-occurrence analysis and thematic mapping, complemented by a factorial analysis of the most cited documents (Figure 6A,B).

The thematic map (Figure 6A) reveals a well-defined conceptual core of the field, dominated by quantitative microbial risk assessment, QMRA, and predictive microbiology. These themes occupy central positions with high relevance and moderate to high development, indicating that they form the methodological backbone of contemporary food safety risk assessment. Their positioning reflects sustained scholarly attention and their role as integrative frameworks connecting microbiological data, exposure modelling, and public health risk characterization.

Closely linked to this core are pathogen-specific themes, particularly *Campylobacter*, *Salmonella*, *Listeria monocytogenes*, and *Escherichia coli*. *Campylobacter* and microbial risk assessment appear as highly developed and central themes, reflecting their long-standing importance in foodborne disease burden assessments, especially in poultry and meat supply chains. In contrast, *Escherichia coli* and *QMRA* appear in the basic themes quadrant, suggesting that while these topics are fundamental to the field, their conceptual development continues to rely on established modelling approaches rather than novel theoretical expansion.

Several applied and process-oriented themes, such as cross-contamination, food safety, and dose–response, are positioned in the lower-central or emerging quadrants. This indicates either specialised application contexts or topics that are currently under renewed investigation due to changing production systems, novel foods, or evolving regulatory requirements. Notably, dose–response modelling appears as an emerging or declining theme, highlighting a persistent methodological challenge where data limitations continue to constrain progress despite its central importance for quantitative risk estimation.

The factorial map of the most cited documents (Figure 6B) further contextualises these thematic patterns by revealing distinct intellectual clusters underpinning the field. Seminal works published in the *International Journal of Food Microbiology* and *Preventive Veterinary Medicine* form one major axis, focusing on pathogen behaviour, exposure pathways, and early quantitative modelling approaches. Another prominent cluster is anchored in methodological and statistical contributions, particularly those addressing uncertainty analysis, Monte Carlo simulation, and probabilistic modelling frameworks. These clusters collectively illustrate how QMRA research has evolved through the interaction of microbiology-driven case studies and advances in quantitative risk modelling techniques.

Importantly, the spatial separation between clusters suggests that while methodological innovation and applied pathogen-specific studies are strongly connected, greater integration between advanced statistical methods and emerging application domains, such as environmental pathways, wastewater reuse, and complex food systems, represents a key opportunity for future research.

Overall, RQ5 highlights a field that is methodologically mature but thematically dynamic. Core QMRA concepts and predictive microbiology remain central, while future research directions are likely to focus on improving dose–response modelling, integrating uncertainty and variability more explicitly, and expanding QMRA applications beyond traditional food matrices to encompass environmental and system-level food safety challenges.

### 3.6. Limitations

This study is subject to several inherent limitations. The analysis was based exclusively on records retrieved from the Scopus database. Although Scopus provides broad coverage and standardized metadata suitable for bibliometric analysis, relevant studies indexed in other databases or present in grey literature may not have been captured. In addition, the search strategy prioritised explicit use of quantitative and probabilistic risk assessment terminology to ensure conceptual consistency, which may have excluded studies applying QMRA principles implicitly or using alternative terminology. In addition, the restriction to English-language publications may have introduced language bias and led to the underrepresentation of research conducted in non-English-speaking countries. Although many internationally visible journals publish in English, QMRA studies are also disseminated through national and regional journals in local languages, particularly in countries with strong domestic research communities. Consequently, relevant contributions from certain geographic regions and research traditions may not have been fully captured, potentially influencing the observed distribution of publication output, collaboration networks, and thematic patterns.

The included body of evidence reflects substantial heterogeneity in modelling approaches, data sources, and reporting practices across QMRA studies, which limits direct comparability between studies. Differences in model structure, parameterisation, and assumptions further constrain the transferability of findings across food–pathogen contexts.

Bibliometric indicators primarily reflect publication activity, citation visibility, and structural relationships rather than the methodological quality or regulatory impact of individual studies. Citation-based metrics are also influenced by publication age, potentially underestimating the contribution of more recent research, and may additionally be affected by self-citation practices that can inflate perceived influence.

Furthermore, as a bibliometric study focused on structural characteristics of the literature rather than synthesis of effect-based evidence, no formal risk of bias assessment, effect size synthesis, or certainty of evidence evaluation was conducted. These methodological features should be considered when interpreting the findings. Despite these limitations, the dataset captures the core structure and dominant trends of QMRA research and provides a robust and reproducible overview of the field’s evolution, collaboration patterns, and thematic development.

## 4. Conclusions

This bibliometric analysis offers a concise quantitative overview of the evolution, structure, and thematic focus of quantitative microbial risk assessment (QMRA) research in food safety. The results show that QMRA has developed as a methodologically mature field with steady growth over time, reflecting the technical complexity and substantial data requirements inherent to probabilistic microbial risk modelling. Rather than rapid expansion, recent publication trends indicate consolidation and refinement of established approaches, suggesting that methodological progress is increasingly incremental rather than transformative.

The influence structure of the field is clearly concentrated. A limited number of specialised journals, research institutions, and author groups account for a substantial share of publications and citations, highlighting stable methodological leadership and continuity. This concentration supports the development of shared modelling practices and contributes to the comparability of QMRA applications across different food–pathogen contexts, but may also limit methodological diversity and reduce the visibility of alternative modelling approaches and region-specific risk scenarios.

Geographically, QMRA research is dominated by Europe and North America, with international collaboration playing a central role, particularly within European research networks. The high proportion of multi-country publications underscores the transnational nature of QMRA, driven by shared data, regulatory frameworks, and collaborative risk assessment initiatives. However, the limited representation of low- and middle-income regions suggests that globally diverse food systems, informal supply chains, and region-specific exposure patterns remain insufficiently addressed in the current evidence base.

Thematic analysis confirms that QMRA remains anchored in probabilistic exposure assessment, Monte Carlo simulation, predictive microbiology, and dose–response modelling, with research predominantly focused on major foodborne pathogens such as *Campylobacter*, *Salmonella*, *Listeria monocytogenes*, and pathogenic *Escherichia coli*. While this reflects methodological consolidation, it also indicates slower expansion toward underexplored hazards, emerging food production systems, and complex environmental transmission pathways. At the same time, recurring emphasis on uncertainty, cross-contamination, and dose–response relationships highlights persistent methodological challenges and areas where further harmonisation and data improvement are needed.

Overall, the findings depict QMRA as a coherent and well-established research domain that continues to evolve incrementally. Beyond mapping the structure of the field, this bibliometric overview highlights the need for broader geographic inclusion, greater methodological diversification, and stronger integration of emerging data sources in order to enhance the global relevance and future adaptability of QMRA in food safety decision-making.

## Data Availability

The bibliographic dataset generated and analysed during the current study is available from the corresponding author upon reasonable request. The search strategy, inclusion criteria, and PRISMA materials are provided in the Supplementary Materials.

## Supplementary Materials

The following supporting information can be downloaded at: https://www.mdpi.com/article/10.3390/foods15071197/s1. Supplementary Materials: File S1: PRISMA 2020 checklist; Table S1: List of included studies (*n* = 186) with bibliographic characteristics.

## Abbreviations

The following abbreviations are used in this manuscript:

QMRA	Quantitative Microbial Risk Assessment
RIVM	National Institute for Public Health and the Environment
DTU	Technical University of Denmark
EFSA	European Food Safety Authority
STEC	Shiga toxin–producing *Escherichia coli*
MCP	Multi-Country Publications
SCP	Single-Country Publications
