# Supplementary material for: A Bibliometric and Systematic Review of Quantitative Microbial Risk Assessment in Food Safety (1995–2024)
Source: Foods. 2026 Apr 2;15(7):1197. doi: 10.3390/foods15071197 (PMC13074090; doi:10.3390/foods15071197)
Supplement: Supplementary file 1 [file foods-15-01197-s001.zip › foods-4186632-supplementary/Suplementary/File S1. PRISMA_2020_checklist.pdf]

PRISMA 2020 Checklist

Section and Topic	Item #	Checklist item	Location where item is reported
**TITLE**			
Title	1	Identify the report as a systematic review.	Title, Page 1
**ABSTRACT**			
Abstract	2	See the PRISMA 2020 for Abstracts checklist.	Abstract, Page 1
**INTRODUCTION**			
Rationale	3	Describe the rationale for the review in the context of existing knowledge.	Introduction, Page 2,3
Objectives	4	Provide an explicit statement of the objective(s) or question(s) the review addresses.	Introduction, Page 2,3
**METHODS**			
Eligibility criteria	5	Specify the inclusion and exclusion criteria for the review and how studies were grouped for the syntheses.	Materials and Methods, Data screening and inclusion criteria
Information sources	6	Specify all databases, registers, websites, organisations, reference lists and other sources searched or consulted to identify studies. Specify the date when each source was last searched or consulted.	Materials and Methods, Data source and search strategy (Scopus database; search conducted on January 24, 2026)
Search strategy	7	Present the full search strategies for all databases, registers and websites, including any filters and limits used.	Materials and Methods, Data source and search strategy (full search query provided)
Selection process	8	Specify the methods used to decide whether a study met the inclusion criteria of the review, including how many reviewers screened each record and each report retrieved, whether they worked independently, and if applicable, details of automation tools used in the process.	Materials and Methods, PRISMA workflow description (independent screening by first and senior author; consensus-based resolution)
Data collection process	9	Specify the methods used to collect data from reports, including how many reviewers collected data from each report, whether they worked independently, any processes for obtaining or confirming data from study investigators, and if applicable, details of automation tools used in the process.	Materials and Methods, Data source and

PRISMA

PRISMA 2020 Checklist

Section and Topic	Item #	Checklist item	Location where item is reported
			search strategy (data export from Scopus and preparation procedures)
Data items	10a	List and define all outcomes for which data were sought. Specify whether all results that were compatible with each outcome domain in each study were sought (e.g. for all measures, time points, analyses), and if not, the methods used to decide which results to collect.	Not applicable (bibliometric analysis; no outcome measures extracted)
	10b	List and define all other variables for which data were sought (e.g. participant and intervention characteristics, funding sources). Describe any assumptions made about any missing or unclear information.	Materials and Methods, Data source and search strategy (bibliographic variables: authors, affiliations, source titles, publication year, citations, and keywords)
Study risk of bias assessment	11	Specify the methods used to assess risk of bias in the included studies, including details of the tool(s) used, how many reviewers assessed each study and whether they worked independently, and if applicable, details of automation tools used in the process.	Not applicable (no risk of bias assessment conducted due to bibliometric study design)
Effect measures	12	Specify for each outcome the effect measure(s) (e.g. risk ratio, mean difference) used in the synthesis or presentation of results.	Not applicable (no effect size estimation performed)
Synthesis methods	13a	Describe the processes used to decide which studies were eligible for each synthesis (e.g. tabulating the study intervention characteristics and comparing against the planned groups for each synthesis (item #5)).	Materials and Methods, Data screening and inclusion criteria (all included studies analysed collectively in bibliometric framework)
	13b	Describe any methods required to prepare the data for presentation or synthesis, such as handling of missing summary statistics, or data conversions.	Materials and Methods, Data source and

Section and Topic	Item #	Checklist item	Location where item is reported
			search strategy (data standardization and cleaning procedures)
	13c	Describe any methods used to tabulate or visually display results of individual studies and syntheses.	Materials and Methods, Bibliometric analysis (network visualizations and thematic mapping)
	13d	Describe any methods used to synthesize results and provide a rationale for the choice(s). If meta-analysis was performed, describe the model(s), method(s) to identify the presence and extent of statistical heterogeneity, and software package(s) used.	Materials and Methods, Bibliometric analysis (performance analysis and science mapping using Bibliometrix in R)
	13e	Describe any methods used to explore possible causes of heterogeneity among study results (e.g. subgroup analysis, meta-regression).	Not applicable (no meta-analysis or subgroup analysis performed)
	13f	Describe any sensitivity analyses conducted to assess robustness of the synthesized results.	Not applicable (no sensitivity analyses conducted)
Reporting bias assessment	14	Describe any methods used to assess risk of bias due to missing results in a synthesis (arising from reporting biases).	Not applicable (reporting bias not assessed in bibliometric analysis)
Certainty assessment	15	Describe any methods used to assess certainty (or confidence) in the body of evidence for an outcome.	Not applicable (certainty of evidence not evaluated)
**RESULTS**			
Study selection	16a	Describe the results of the search and selection process, from the number of records identified in the search to the number of studies	Materials and

Section and Topic	Item #	Checklist item	Location where item is reported
		included in the review, ideally using a flow diagram.	Methods (PRISMA description); Results and Discussion (first paragraph); Supplementary Figure S1
	16b	Cite studies that might appear to meet the inclusion criteria, but which were excluded, and explain why they were excluded.	No studies excluded after screening; exclusions only via predefined filters (Methods)
Study characteristics	17	Cite each included study and present its characteristics.	Results and Discussion (first paragraph – general characteristics); full listing not provided (bibliometric scope)
Risk of bias in studies	18	Present assessments of risk of bias for each included study.	Bibliometric study – no individual study quality assessment performed
Results of individual studies	19	For all outcomes, present, for each study: (a) summary statistics for each group (where appropriate) and (b) an effect estimate and its precision (e.g. confidence/credible interval), ideally using structured tables or plots.	No effect-size synthesis; bibliometric analysis only
Results of syntheses	20a	For each synthesis, briefly summarise the characteristics and risk of bias among contributing studies.	Results and Discussion (dataset description and thematic overview); risk of bias not assessed
	20b	Present results of all statistical syntheses conducted. If meta-analysis was done, present for each the summary estimate and its precision (e.g. confidence/credible interval) and measures of statistical heterogeneity. If comparing groups, describe the direction of the effect.	Results and Discussion (RQ1–RQ4; bibliometric

Section and Topic	Item #	Checklist item	Location where item is reported
			indicators, trends, networks)
	20c	Present results of all investigations of possible causes of heterogeneity among study results.	Not applicable (no meta-analysis)
	20d	Present results of all sensitivity analyses conducted to assess the robustness of the synthesized results.	Not applicable
Reporting biases	21	Present assessments of risk of bias due to missing results (arising from reporting biases) for each synthesis assessed.	Not assessed (bibliometric scope)
Certainty of evidence	22	Present assessments of certainty (or confidence) in the body of evidence for each outcome assessed.	Not assessed (no outcome-based synthesis)
**DISCUSSION**			
Discussion	23a	Provide a general interpretation of the results in the context of other evidence.	Results and Discussion (RQ1–RQ4 interpretative sections; especially synthesis paragraphs after each RQ)
	23b	Discuss any limitations of the evidence included in the review.	Limitations section (paragraph on heterogeneity, variability in modelling approaches, and comparability constraints)
	23c	Discuss any limitations of the review processes used.	Limitations section (Scopus-only database, search strategy constraints, terminology bias)
	23d	Discuss implications of the results for practice, policy, and future research.	Conclusions section (final paragraphs on methodological refinement, regulatory relevance, and

PRISMA

PRISMA 2020 Checklist

Section and Topic	Item #	Checklist item	Location where item is reported
			future research directions)
**OTHER INFORMATION**			
Registration and protocol	24a	Provide registration information for the review, including register name and registration number, or state that the review was not registered.	This review was not registered.
	24b	Indicate where the review protocol can be accessed, or state that a protocol was not prepared.	No formal review protocol was prepared prior to conducting this study.
	24c	Describe and explain any amendments to information provided at registration or in the protocol.	Not applicable, as the review was not registered and no protocol was prepared.
Support	25	Describe sources of financial or non-financial support for the review, and the role of the funders or sponsors in the review.	This research received no external funding. The authors declare that no funders had any role in the design of the study; in the collection, analysis, or interpretation of data; or in the writing of the manuscript.
Competing interests	26	Declare any competing interests of review authors.	The authors declare no conflict of interest.
Availability of data, code and other materials	27	Report which of the following are publicly available and where they can be found: template data collection forms; data extracted from included studies; data used for all analyses; analytic code; any other materials used in the review.	The data supporting this study are derived from the Scopus database. Extracted bibliographic data and analysis scripts (e.g., bibliometrix in R) are available

PRISMA 2020 Checklist

Section and Topic	Item #	Checklist item	Location where item is reported
			from the corresponding author upon reasonable request.

From: Page MJ, McKenzie JE, Bossuyt PM, Boutron I, Hoffmann TC, Mulrow CD, et al. The PRISMA 2020 statement: an updated guideline for reporting systematic reviews. BMJ 2021;372:n71. doi: 10.1136/bmj.n71. This work is licensed under CC BY 4.0. To view a copy of this license, visit https://creativecommons.org/licenses/by/4.0/
